# Metasurface interferometry toward quantum sensors

**DOI:** 10.1038/s41377-019-0182-6

**Published:** 2019-08-14

**Authors:** Philip Georgi, Marcello Massaro, Kai-Hong Luo, Basudeb Sain, Nicola Montaut, Harald Herrmann, Thomas Weiss, Guixin Li, Christine Silberhorn, Thomas Zentgraf

**Affiliations:** 1 Paderborn University, Department of Physics, Warburger Str. 100, 33098 Paderborn, Germany; 2 University of Stuttgart, 4th Physics Institute, Pfaffenwaldring 57, 70569 Stuttgart, Germany; 3 Southern University of Science and Technology, Department of Materials Science and Engineering, Shenzhen Institute for Quantum Science and Engineering, 518055 Shenzhen, China

**Keywords:** Metamaterials, Single photons and quantum effects, Quantum optics

## Abstract

Optical metasurfaces open new avenues for the precise wavefront control of light for integrated quantum technology. Here, we demonstrate a hybrid integrated quantum photonic system that is capable of entangling and disentangling two-photon spin states at a dielectric metasurface. Via the interference of single-photon pairs at a nanostructured dielectric metasurface, a path-entangled two-photon NOON state with circular polarization that exhibits a quantum HOM interference visibility of 86 ± 4% is generated. Furthermore, we demonstrate nonclassicality andphase sensitivity in a metasurface-based interferometer with a fringe visibility of 86.8 ± 1.1% in the coincidence counts. This high visibility proves the metasurface-induced path entanglement inside the interferometer. Our findings provide a promising way to develop hybrid-integrated quantum technology operating in the high-dimensional mode space in various applications, such as imaging, sensing, and computing.

## Metasurfaces: An entangled path to hybrid quantum photonic technologies

Scientists have developed an optical metasurface capable of entangling and disentangling photon-pairs, providing a path for the development of quantum technologies for applications in computing, imaging, and sensing. Optical metasurfaces are sub-wavelength layers of nanostructures capable of precisely controlling the properties of light. They offer the promise of new miniaturized quantum systems where they remain largely unexplored. Now Thomas Zentgraf and colleagues from the University of Paderborn in Germany, working with researchers from the University of Stuttgart and the Southern University of Science and Technology in China, have developed a nanostructured dielectric metasurface capable of entangling and disentangling the spin states of a photon-pair. Quantum interference of the photons on the metasurface produces a circularly polarized entangled photon-pair, which can be disentangled by passing it through the metasurface a second time.

## Introduction

Integrated quantum technology is widely used to enable quantum applications in various systems for secure quantum communication^1–3^ as well as in quantum simulation^4–7^ and quantum metrology^8^. Although significant progress in terms of large-scale integration in one generic material has been achieved^9^, highly miniaturized integrated systems are required for more complex functionalities, such as teleporting twisted photons^10^ in a high-dimensional spin–orbital angular momentum (OAM) space^11^. However, the on-chip manipulation of circularly polarized photons is still an unsolved problem.

State-of-the-art metasurfaces essentially achieve any kind of manipulation of light wavefronts for applications such as ultra-flat lenses for imaging^12,13^, vector beam generation^14,15^, optical holography^16^, and even nonlinear phase manipulation^17,18^. These diverse functionalities can be achieved by tailoring the local geometries of the nanostructured surfaces. While all these concepts solely rely on the classical electromagnetic description, the potential of metasurfaces for quantum applications is still widely unexplored. However, as versatile optical elements for locally altering the amplitude, phase, and polarization of light^19–21^, metasurfaces can provide new functionalities to miniaturized quantum systems.

Recently, few initiatives have been taken to investigate the potential of metasurfaces in quantum optics. Jha et al.^22,23^ theoretically proposed that a metasurface can induce quantum interference between orthogonal radiative transition states of atoms and quantum entanglement between two qubits. Later, it was demonstrated that the entanglement of the spin and orbital angular momentum of a single photon can be generated via a metasurface^24^. In addition, the metasurface can provide a compact solution for quantum state reconstruction^25^. However, thus far, there is only limited experimental evidence revealing whether metasurfaces are suited tostate manipulation in quantum optical experiments. If the current metasurface technology can be directly applied to practical quantum applications, then metasurfaces can offer advanced solutions for quantum imaging^26,27^, sensing^28^, and computing^29^.

Here, we demonstrate the entanglement and disentanglement of two-photon states using an all-dielectric metasurface. Our metasurface allows the generation of path-entangled NOON states with circular polarization due to the quantum interference effect. We observe photon bunching within two spatially distinct output channels of the metasurface. Passing the same metasurface the second time, the generated path-entangled two-photon spin state can be disentangled without introducing additional phase information. Our experiments indicate that metasurfaces are perfectly suited to provide large-scale and high-dimensional quantum functionalities with properties that go far beyond the conventional optical elements. Thus, the hybrid integration of quantum optical elements together with metasurfaces offers the potential to deliver robust multiphoton entanglement and high-dimensional quantum applications, which allows to encode more information in the high-dimensional domain by combining OAM with hybrid space-polarization entanglement^30^.

## Results

### Metasurface design and functionality

For the experiment, we designthe metasurface to deflect the incident light into two different output directions under angles of ±10°. The deflection is obtained by using a space-variant Pancharatnam-Berryphase that results from the polarization conversion of the transmitted light^31^. As a platform for the metasurface, we use silicon nanofin structures. They act as local halfwave plates, which convert the circular polarization states into their cross polarization and add a spatial phase term based on their orientation angle. For our design, we choose a linear phase gradient, which diffracts the incoming light under the desired angle. We note that the sign of the phase gradient explicitly depends on the helicity of the circularly polarized light, which effectively makes our metasurface a spatial separator for the circular polarization states (for more details, see Supplementary Information).

### Entanglement and disentanglement

**Fig. 1:**
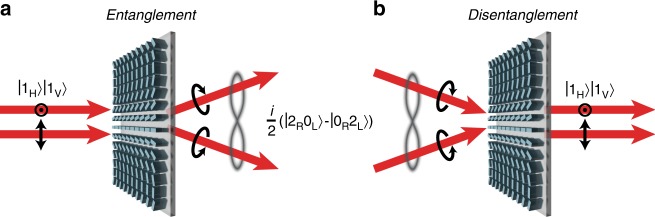
Spatial entanglement and disentanglement of a two-photon state at a metasurface. **a** If a single photon pair with orthogonal linear polarization is inserted into the metasurface, it is divided into its circular polarization components. Since the inserted quantum state is a two-photon NOON state in the circular polarization basis, both photons will always choose the same output channel, and spatial entanglement is obtained. **b** The situation reverses for the insertion of a spatially entangled NOON state into the metasurface. The quantum interference leads to a projection back to the original orthogonal linear polarization states, and the photon pair is spatially disentangled

Precise control and preparation of multiphoton entanglement are of fundamental interest for quantum technologies. With the accurate manipulation of the single photons’ wavefront by a metasurface, spatial and polarization-based entanglement can be achieved. In our case, the metasurface is designed to spatially separate the generated circular polarization states of light and thus enforces a quantum state’s representation in that particular basis, as sketched in Fig. 1a. For the particular quantum state $|{\mathrm{\Psi }}\rangle = \hat a_H^\dagger \hat a_V^\dagger |0 \rangle$, such a process at the metasurface leads to an entangled state. Note that we use the indices *H* and *V* for the two linear polarizations states and *L* and *R* for the circular polarization states. Starting with relations between the photon creation operators

1 $$\begin{array}{l}\hat a_L^\dagger = \frac{1}{{\sqrt 2 }}\left( {\hat a_H^\dagger + i \cdot \hat a_V^\dagger } \right)\\ \hat a_R^\dagger = \frac{1}{{\sqrt 2 }}\left( {\hat a_H^\dagger - i \cdot \hat a_V^\dagger } \right)\end{array}$$

the metasurface enforces a change in the initial quantum state $|{\mathrm{\Psi }}\rangle$ in the circular basis as

2 $$|{\mathrm{\Psi }} \rangle = \hat a_H^\dagger \hat a_V^\dagger |0\rangle = - \frac{i}{2}\left( {\hat a_L^\dagger \hat a_L^\dagger - \hat a_R^\dagger \hat a_R^\dagger } \right)|0\rangle$$

which corresponds to a two-photon NOON state. In general, NOON states are entangled N-photon quantum states, which are commonly used for quantum metrology. Formally, these states can be written as a superposition of two quantum states with all photons allocated in only one of the two channels^32^

3 $$| {{\mathrm{\Psi }}_{{\mathrm{NOON}}}} \rangle = \frac{1}{{\sqrt 2 }}\left( {| N\rangle |0\rangle + |0\rangle| N \rangle } \right)$$

Through metasurface-induced spatial separation of the two polarization channels, this path-encoded quantum state cannot be decomposed into two single-photon states and is therefore path-entangled.

Interestingly, the same metasurface can be used in the reverse process, in which the circular polarization states are spatially recombined to disentangle the generated state. We utilize the polarization basis change functionality to build a metasurface-based interferometer. By introducing a phase delay *φ* between the two circular polarization channels and using the conversion of the polarization states of the metasurface, we obtain the quantum state

4 $$\begin{array}{l}|{\mathrm{\Psi }} \rangle _\varphi = - \frac{i}{2}\left( {\hat a_L^\dagger \hat a_L^\dagger e^{ - i2\varphi } - \hat a_R^\dagger \hat a_R^\dagger } \right)| 0 \rangle \\ = \frac{1}{2}\left( {e^{ - i2\varphi } + 1} \right)\hat a_H^\dagger \hat a_V^\dagger |0\rangle - \frac{i}{4}\left( {e^{ - i2\varphi } - 1} \right)\left( {\hat a_H^\dagger \hat a_H^\dagger - \hat a_V^\dagger \hat a_V^\dagger } \right)| 0 \rangle \end{array}$$

For the case where $\varphi = {\mathrm{n\pi }},\,({\mathrm{n}}\epsilon {\Bbb Z})$, the output state after passing through the metasurface twice is the same as the initial two-photon state $\hat a_H^\dagger \hat a_V^\dagger |0\rangle$, which is disentangled (as shown in Fig. 1b). In the case of $\varphi = \left( {{\mathrm{n}} + \frac{1}{2}} \right){\mathrm{\pi }},\,({\mathrm{n}}\epsilon {\Bbb Z})$, the output state $i \cdot \frac{1}{2} ( {\hat a_H^\dagger \hat a_H^\dagger - \hat a_V^\dagger \hat a_V^\dagger } )|0\rangle$ is entangled even after spatial recombination, as it cannot be decomposed in either polarization base.

### Generation of NOON spin states

**Fig. 2:**
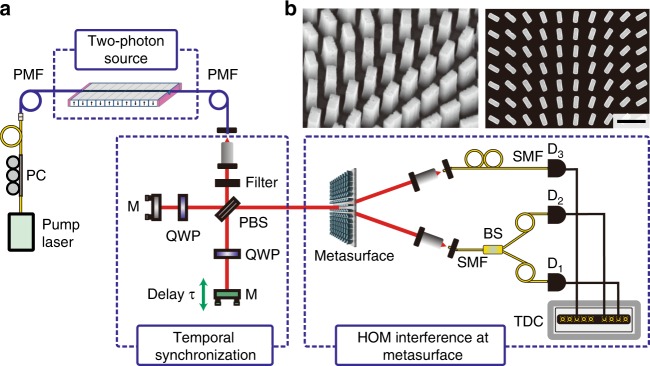
Illustration of the measurement setup. **a** The two-photon source creates a photon pair with orthogonal linear polarizations by spontaneous parametric down-conversion (SPDC). The two photons are temporally delayed relative to each other by *τ* with a Michelson interferometer containing a polarizing beam splitter (PBS) and quarter-wave plates (QWPs). The photon pair passes through the metasurface where the NOON state is generated. The spatially entangled state is then analyzed by a coincidence measurement system in two different configurations with single photon detectors (*D*_*n*_). PMF polarization-maintaining fiber, SMF single mode fiber, M mirror, PC polarization controller, TDC time-digital converter. **b** Scanning electron microscopy images at 45° (left) and the top view (right) for a small area of the fabricated silicon metasurface (scale bar: 1 µm)

First, we investigate the NOON-state generation at the metasurface. A key quantum feature of the NOON state is its photon bunching characteristics, i.e., we expect that both photons will always choose the same metasurface output channel after inserting the quantum state $\hat a_H^\dagger \hat a_V^\dagger | 0 \rangle$. To demonstrate this bunching effect, we use the setup shown in Fig. 2. It contains four logical parts: a two-photon source for the generation of the initial quantum state $|1_H\rangle|1_V\rangle$, a Michelson interferometer to adjust the time delay between these two photons, a metasurface as a quantum interference device, and a single-photon detection system. To control the time delay between the two photons, we use a modified Michelson interferometer with a PBS cube and two quarter-wave plates. Since the time delay directly influences the temporal overlap between both photons, we utilize it to enable $(\tau = 0)$ and disable $(\tau \to \infty )$ the quantum interference effect at the metasurface. In the case of no interference, the two photons will choose either output with a 50% chance, while in the case of perfect interference, both photons will always choose the same random output port.

To characterize the generated quantum state, we perform two different coincidence measurements for various time delays *τ*. First, we measure the number of coincidences between both output channels of the metasurface. The generated NOON state should not contribute to the number of registered coincidences, ideally leading to zero coincidence counts. However, when the initial $|1_H\rangle$ and $|1_V\rangle$ photons do not arrive simultaneously at the metasurface, we expect a 50% coincidence probability per inserted photon pair (without losses). Thus, as we vary the time delay *τ*, we expect the well-known Hong-Ou-Mandel (HOM) correlation dip. Note that a visibility higher than 50% verifies the quantum character of interference for these HOM experiments^33^. Second, we measure the number of coincidences between the two outputs of a 50:50 beam splitter that has been placed in one of the output channels of the metasurface. Coincidence counts for this measurement can be obtained only when both photons choose the same output channel of the metasurface. Since the probability for this event is twice as large in the case of interference, we expect a 2:1 ratio in the coincidence counts between *τ* = 0 and $\tau \to \infty$. Both coincidence measurements are performed simultaneously by using three superconducting nanowire single-photon detectors. One of these detectors (*D*_3_) is directly connected to one of the metasurface output channels, while the other two detectors (*D*_1_ and *D*_2_) are placed behind an integrated beam splitter (a 3-dB fiber coupler), which is connected to the second metasurface output channel. In this configuration, the first coincidence measurement between the two metasurface channels can be calculated as $C_{13} + C_{23}$, where *C*_*ij*_ refers to the coincidence between the detectors *D*_*i*_ and *D*_*j*_.

**Fig. 3:**
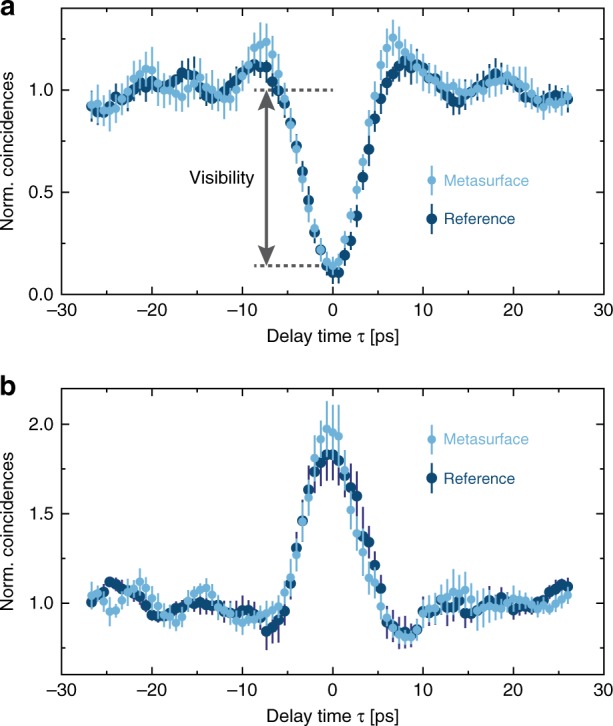
Hong-Ou-Mandel coincidence measurements. **a** Normalized coincidence counts $\left( {{C}_{13} + {C}_{23}} \right)$ between the two output channels of the metasurface for a variation of the initial photon time delay. The high visibility beyond the classical limit of 50% confirms the expected quantum interference effect. The reference is obtained for a standard Hong-Ou-Mandel experiment with a standard beam splitter to characterize the quality of the photon source. **b** Normalized coincidence counts *C*_12_ between detectors *D*_1_ and *D*_2_ for the measurement with the polarization beam splitter in one output channel of the metasurface. The peak in the coincidence counts of the “anti”-HOM measurement confirms that the two photons always take the same output channel

The coincidences show a clear dip at zero-time delay with a high visibility of 86 ± 4% (Fig. 3a). Note that the visibility clearly exceeds the limit of 50%, which can be achieved with classical coherent light. At the same time, the second coincidence measurement (*C*_12_) shows a clear coincidence peak (“anti”-HOM peak) at *τ* = 0, which confirms that the two photons are always bunched together in one output channel (Fig. 3b). In the case of interference, both measurements show that the probability of at least one photon in each output channel decreases (first measurement), while the probability of at least two photons in one output channel increases (second measurement). Thus, photon bunching occurs (for details, see Supplementary Information).

### Metasurface-based interferometer

**Fig. 4:**
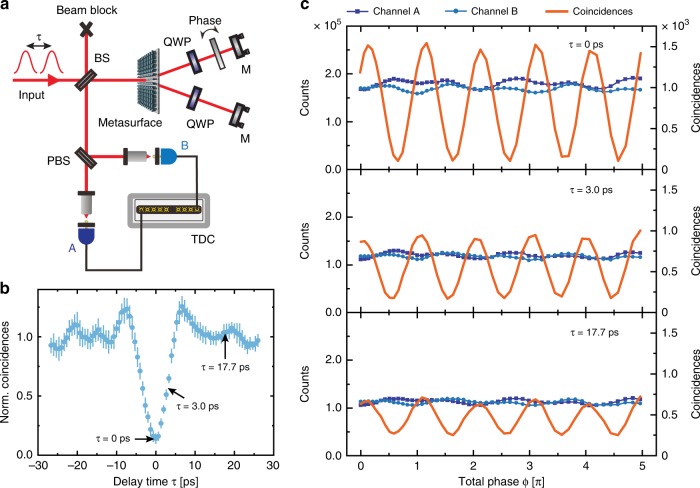
Quantum measurements on a metasurface-based interferometer. **a** Schematic view of the metasurface-based interferometer using a time delay *τ* between the two input photons. **b** Selected time delays *τ* illustrated at the HOM-dip measurement. **c** Experimental results for the two-photon state with three different time delays *τ* after passing through the MBI. The count rates at both detectors are independent of the introduced phase *ϕ* and the time delay *τ*. The measured coincidence rates show an oscillatory behavior with lower visibility at higher delaytimes. The obtained visibility values are 86.8 ± 1.1% for the case of no time delay, 67 ± 2% for $\tau = 3.0\,{\mathrm{ps}}$ and 44 ± 5% for $\tau = 17.7\,{\mathrm{ps}}$

Next, we study whether the metasurface also preserves the quantum coherence of the generated state, i.e., the phase relations between the components of the superposition are fixed and not randomly redistributed. Such coherence is important in quantum metrology applications, where phase measurements play a key role. For that, we realized a folded metasurface-based interferometer (MBI) in which the photons pass through the metasurface twice (Fig. 4a). By tilting a 130-µm-thick glass plate in one of the two arms of the interferometer, we introduce a phase difference between the two optical paths. The final state is then separated and analyzed at a polarizing beam splitter (PBS).

First, we use a strongly attenuated laser as a weak coherent input state. For the input state $\left| \alpha \right._H\rangle\left| 0 \right._V\rangle$, i.e., a coherent state in one input and vacuum in the other, the count rates in both output ports of the MBI show an oscillating behavior. At the same time, the coincidences show an oscillating behavior with the doubled frequency following a ${\mathrm{sin}}^2\left( \varphi \right)$ function (for details, see Fig. S2b of the Supplementary Information). This behavior can be understood from a classical point of view, where the coincidence corresponds to the product of the single counts. The fringe contrast of 90 ± 1% is in good agreement with our theoretical estimations (see Supplementary Information). Note that *φ* corresponds to the introduced total phase by passing twice through the tilted glass plate.

Next, we launch one photon per input mode $\left| 1 \right._H\rangle\left| 1 \right._V\rangle$ by using the photon pairs from the SPDC source. We observe that the counts at each individual MBI output channel will be constant regardless of the introduced phase *φ* (Fig. 4c). This is due to the first-order correlation between two MBI outputs being independent of *φ* (see Supplementary Information). However, when we determine the coincidences between the two outputs, we observe the same double frequency oscillation from the coherent case, which is now phase shifted according to a ${\mathrm{cos}}^2\left( \varphi \right)$ function. When two orthogonally polarized photons arrive at the metasurface simultaneously ($\tau = 0$), there is no coincidence contribution in the HOM experiment due to the photon bunching effect (as shown in Fig. 4b). In this scenario, path-entangled photon pairs are generated at the metasurface. Thus, the visibility of interference fringes from the MBI is $86.8 \pm 1.1\%$, which is beyond the violation limit of Bell’s inequalities ($70.7\%$). In addition, further experiments for various time delays *τ* between the initial two photons show a reduced visibility of the coincidence rate (see Fig. 4c). When one photon is delayed by 3 ps, the normalized HOM coincidence rate of approximately 50% tells us that the two photons still overlap partially in time. For this partial overlap, the visibility of the interference fringes is 67 ± 2%, which is close to the boundary of Bell’s inequalities. If one photon is delayed by more than 17 ps, they arrive at the MBI one after the other. Correspondingly, no path entanglement is generated inside the MBI. This is in good agreement with our experimental visibility of $45 \pm 5\%$ for the coincidence rate.

In contrast to the coherent case, the coincidence counts can no longer be perceived as the product of the single counts. This pure nonclassical effect is a key feature of quantum interferometry that is closely related to photon entanglement. The peaks in the coincidences result from the second-order correlation of the entangled NOON spin state with circular polarization, which is generated after passing through the metasurface the first time. Passing through the entire MBI, the entangled NOON spin state is disentangled into the original two-photon state $\hat a_H^\dagger \hat a_V^\dagger |0\rangle$ if there is no phase difference between two MBI arms (first term in Eq. (4)). After splitting the state at the PBS, the two photons arrive at the two detectors simultaneously, which causes the maximum coincidence rates. On the other hand, the minima in the coincidences result from the second-order correlation of the entangled NOON state with linear polarizations (second term in Eq. (4)). For phase differences of $\varphi = \left( {{n} + \frac{1}{2}} \right){\mathrm{\pi }},\,({n}\epsilon {\Bbb Z})$, the output of the MBI is solely determined by the second term of Eq. (4). The two photons are either in the *H* or *V* path, but they cannot be in both paths simultaneously. Hence, there is no coincidence contribution, similar to the HOM effect, as shown in Fig. 3.

## Discussion

To quantify the quality of the quantum interference and the entanglement that takes place at the metasurface, we compare the visibility of the HOM dip obtained for the metasurface with a reference experiment. The reference experiment is inspired by Grice and Walmsley^33^ and allows us to determine an upper bound on the achievable HOM visibility, which is approximately $89 \pm 5\%$ (for more details, see Supplementary Information). The visibility of the HOM dip, which depends not only on the quality of the SPDC source but also on the interference strength of the used interference device, clearly surpasses the classical threshold of 50%. Hence, the high HOM visibility of $86 \pm 4\%$ shows the quantum mechanical nature of the measured spatially entangled two-photon NOON state generated at the metasurface.

The phase information inside the MBI is hidden when performing an intensity (first-order correlation) measurement at each detector. This phase information can be revealed with the help of coincidence (second-order correlation) measurements between the two detectors. This result is similar to the Franson interferometer that is used for verifying the energy-time entanglement^34^ and for security information coding in quantum cryptography. Furthermore, our metasurface-based interferometer shows the feasibility of quantum sensors based on nanostructured metasurfaces. Note that the double period of the fringes itself is not a quantum signature of the two-photon NOON state since it also appears in the coincidence measurement for the weak coherent state^35^.

Integrated photonic quantum experiments are routinely performed using large-scale optical components such as directional couplers and beam splitters. With our experiments, we demonstrate the preparation of spatial entanglement and disentanglement based on a metasurface in a more compact setting. The results are especially remarkable since the metasurface consists of spatially distributed nanostructured elements with slightly different scattering properties. The experiments confirm that quantum entanglement and interference take place at our dielectric metasurface, while phase-sensitive effects (quantum coherence) are preserved. Our findings demonstrate that metasurfaces can achieve an interference performance similar to that oftraditional optical components and are indeed viable candidates for integrated quantum nanosensors and quantum interferometry.

Here, we focused on a metasurface for entanglement and interference and therefore for state manipulation purposes. Our metasurface operates as one of the basic building blocks of typical photonic quantum circuits, which splits and recombines optical modes in nested interferometers. However, metasurfaces have enormous potential in quantum optics. Their ability to fully control the wavefronts of light can be used to generate multiphoton and high-dimensional entanglement with different spin-OAM. Combining multiple optical functionalities into a single metasurface as an efficient and compact quantum optical device might dramatically improve the performance and even lead to new concepts for practical quantum applications. Since metasurfaces can be used directly at waveguides and fiber-end facets, these hybrid nanophotonics systems for arbitrary basis transformation can be used for robust integrated quantum technologies, from sensor arrays to quantum simulators. In this context, future research has to show whether metasurfaces can directly generate quantum states, such as single photon pairs, in a well-defined and efficient way without the need for an additional source.

## Supplementary information

Supplementary Information

## Data Availability

All data needed to evaluate the conclusions in the paper are present in the paper and/or the Supplementary Information. Additional data related to this paper may be requested from the authors.
