# Supplementary material for: Metasurface interferometry toward quantum sensors
Source: Light Sci Appl. 2019 Aug 14;8:70. doi: 10.1038/s41377-019-0182-6 (PMC6804567; doi:10.1038/s41377-019-0182-6)
Supplement: Supplementary file 1 — Supplementary Information [file 41377_2019_182_MOESM1_ESM.pdf]

Supplementary Information

Metasurface interferometry towards quantum sensors

Philip Georgi[1], Marcello Massaro[1], Kai Hong Luo[1], Basudeb Sain[1], Nicola Montaut[1], Harald Herrmann[1], Thomas Weiss[2], Guixin Li[3], Christine Silberhorn[1], Thomas Zentgraf[1]

[1] Paderborn University, Department of Physics, Warburger Str. 100, 33098 Paderborn, Germany
[2] University of Stuttgart, 4th Physics Institute, Pfaffenwaldring 57, 70569 Stuttgart, Germany
[3] Southern University of Science and Technology, Department of Materials Science and Engineering & Shenzhen Institute for Quantum Science and Engineering, 518055 Shenzhen, China

1. Quantum interference at the metasurface

The quantum interference effect at the gradient metasurface results from a change in the polarization basis. Here, we will provide a more detailed derivation for this phenomenon with regard to the output channels of the metasurface. We start our explanation with the classical relation between the linear and the circular polarization basis of light

$$|L\rangle = \frac{1}{\sqrt{2}}\,(|H\rangle + i|V\rangle)$$
$$|R\rangle = \frac{1}{\sqrt{2}}\,(|H\rangle - i|V\rangle) \tag{1}$$

where $|L\rangle$ ($|R\rangle$) stand for the left (right) circular polarization state and $|H\rangle$ ($|V\rangle$) for the horizontal (vertical) polarization, respectively. This single photon relation can be transferred towards a relation of creation operators, since every single photon state $|1_p\rangle$ with polarization p can be obtained by applying the according creation operator $a_p^\dagger$ onto the vacuum state $|0\rangle$.

$$a_L^\dagger = \frac{1}{\sqrt{2}}\left(a_H^\dagger + i a_V^\dagger\right) \wedge \ a_R^\dagger = \frac{1}{\sqrt{2}}\left(a_H^\dagger - i a_V^\dagger\right)$$
$$\Leftrightarrow \ \hat{a}_H^\dagger = \frac{1}{\sqrt{2}}\left(\hat{a}_L^\dagger + \hat{a}_R^\dagger\right) \wedge \ \hat{a}_V^\dagger = \frac{i}{\sqrt{2}}\left(\hat{a}_R^\dagger - \hat{a}_L^\dagger\right) \tag{2}$$

Using these relations, we can express our initial quantum state $|\Psi\rangle$ in the linear basis

$$|\Psi\rangle = \hat{a}_H^\dagger \hat{a}_V^\dagger |0\rangle = -\frac{i}{2}\left(\hat{a}_L^\dagger + \hat{a}_R^\dagger\right)\left(\hat{a}_L^\dagger - \hat{a}_R^\dagger\right)|0\rangle$$
$$= -\frac{i}{2}\left(\hat{a}_L^\dagger \hat{a}_L^\dagger - \hat{a}_R^\dagger \hat{a}_R^\dagger\right)|0\rangle \tag{3}$$

Note, that the initial quantum state is produced by the type-II parametric down-conversion process and possesses one horizontally and one vertically polarized photon. However, in the circular polarization basis, this quantum state is a superposition of either two left polarized or two right polarized photons.

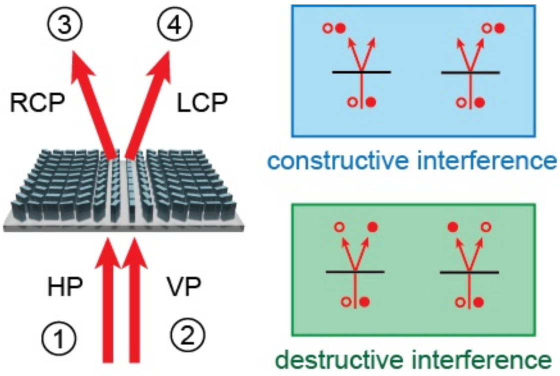

Figure S1. Quantum interference at the metasurface. The dielectric metasurface acts as a circular polarization separator. Here, we explicitly define the two collinear input ports in the linear polarization basis (channel 1&2) and the two-output port in the circular polarization basis (channel 3&4). As a result of the polarization basis change from linear to circular, an interference effect occurs. For one photon in each input channel, we create a path-entangled two-photon NOON state in the output, where both photons will always choose the same output channel.

Based on its design our metasurface has two different functionalities. First, it converts circularly polarized light into its cross-polarization and secondly, it spatially separates the two circular polarization states (Figure S1). The second property is the key feature of our experiment. For any incoming light state, we will only obtain one circular polarization state in each output arm. As such, we can express the creation operators of the four metasurface channels (two input states and two output states) with the creation operators of the four different polarizations for the incoming quantum state:

$$\begin{aligned} \hat{a}_1^\dagger &= \hat{a}_{H,in}^\dagger \\ \hat{a}_2^\dagger &= \hat{a}_{V,in}^\dagger \\ \hat{a}_3^\dagger &= \hat{a}_{R,out}^\dagger = \hat{a}_{L,in}^\dagger \\ \hat{a}_4^\dagger &= \hat{a}_{L,out}^\dagger = \hat{a}_{R,in}^\dagger \end{aligned} \tag{4}$$

Here, the indices of the creation operators correspond to the channel numbers. Note that by inserting Eq. 4 into Eq. 3 we can derive an expression for the quantum state after the metasurface.

$$\begin{aligned} |1{,}1\rangle_{in} = \hat{a}_1^\dagger \hat{a}_2^\dagger |0\rangle &= -\frac{i}{2}\left(\hat{a}_3^\dagger + \hat{a}_4^\dagger\right)\left(\hat{a}_3^\dagger - \hat{a}_4^\dagger\right)|0\rangle \\ &= -\frac{i}{\sqrt{2}}\left(|2{,}0\rangle_{out} - |0{,}2\rangle_{out}\right) \end{aligned} \tag{5}$$

In this Fock-notation, the stated numbers refer to the number of photons in each channel. For one photon in each input channel, we obtain a two-photon NOON state in the output. Since the metasurface spatially separates the circular polarization states, the resulting quantum state is actually entangled, as it cannot be rewritten in the linear polarization basis.

2. Metasurface-based interferometer and phase sensitivity

A metasurface-based interferometer (MBI) is a device composed by a metasurface whose two outputs are fed as inputs to a second metasurface. Between the two metasurfaces, an additional element is placed in one of the two channels in order to introduce a phase delay between the two optical paths.

We label the input modes of the MBI as 1 and 2, while the two output modes are 3 and 4. We will assume that both metasurfaces are lossless. We are looking for a transformation $\mathbf{M}$ that describes the input modes in terms of the output modes, since we are interested in calculating quantities related to the output modes. Because of this, we calculate these relationships "backwards", starting from the final metasurface.

The input modes of the final metasurface $\hat{a}_u^\dagger$ and $\hat{a}_\ell^\dagger$ can be written in terms of the output modes as

$$\begin{pmatrix} \hat{a}_u^\dagger \\ \hat{a}_\ell^\dagger \end{pmatrix} = \mathbf{B} \begin{pmatrix} \hat{a}_3^\dagger \\ \hat{a}_4^\dagger \end{pmatrix} \tag{6}$$

where the subscripts u and ℓ refer to the upper and lower arm of the interferometer and

$$\mathbf{B} = \frac{1}{\sqrt{2}} \begin{pmatrix} 1 & i \\ 1 & -i \end{pmatrix} \tag{7}$$

is the metasurface transformation matrix that satisfying our assumptions. Similarly, the action of the phase shifter on the upper arm can be expressed as

$$\begin{pmatrix} \hat{a}_u^{\dagger\prime} \\ \hat{a}_\ell^{\dagger\prime} \end{pmatrix} = \mathbf{F} \begin{pmatrix} \hat{a}_u^\dagger \\ \hat{a}_\ell^\dagger \end{pmatrix} \tag{8}$$

where $\hat{a}_u^{\dagger\prime}$ and $\hat{a}_\ell^{\dagger\prime}$ are the input modes and

$$\mathbf{F} = \begin{pmatrix} e^{-i\phi} & 0 \\ 0 & 1 \end{pmatrix} \tag{9}$$

is the backward phase-shifter transformation. Lastly, the input modes of the MBI can be expressed in terms of $\hat{a}_u^{\dagger\prime}$ and $\hat{a}_\ell^{\dagger\prime}$

$$\begin{pmatrix} \hat{a}_1^\dagger \\ \hat{a}_2^\dagger \end{pmatrix} = \mathbf{B}^* \begin{pmatrix} \hat{a}_u^{\dagger\prime} \\ \hat{a}_\ell^{\dagger\prime} \end{pmatrix} \tag{10}$$

We can now substitute piece by piece and we find

$$\begin{pmatrix} \hat{a}_1^\dagger \\ \hat{a}_2^\dagger \end{pmatrix} = \mathbf{M} \begin{pmatrix} \hat{a}_3^{\dagger\prime} \\ \hat{a}_4^{\dagger\prime} \end{pmatrix} \tag{11}$$

with the MBI transformation matrix

$$\mathbf{M} = \mathbf{B}^* \cdot \mathbf{F} \cdot \mathbf{B} = \frac{1}{2} \begin{pmatrix} e^{-i\phi} + 1 & i e^{-i\phi} - i \\ -ie^{-i\phi} + i & e^{-i\phi} + 1 \end{pmatrix} \tag{12}$$

If the input is the Fock-state $|1\rangle_1 |1\rangle_2 = \hat{a}_1^\dagger \hat{a}_2^\dagger |0\rangle$, then the output state will be

$$|\Phi\rangle = \left(\frac{1}{2} \hat{a}_3^\dagger \hat{a}_4^\dagger \left(e^{-i2\phi} + 1\right) - \frac{i}{4} \left(\hat{a}_3^{\dagger 2} - \hat{a}_4^{\dagger 2} \right) \cdot \left(e^{-i2\phi} - 1\right) \right) |0\rangle \tag{13}$$

Note, that this expression corresponds to Eq. 3 in our paper. We now calculate the expectation value for the coincidences, defined as

$$\langle \Phi | \hat{n}_3 \hat{n}_4 | \Phi \rangle = \cos^2(\phi) \tag{14}$$

On the other hand, we note that

$$\begin{aligned} \langle \Phi | \hat{n}_3 | \Phi \rangle &= 1 \\ \langle \Phi | \hat{n}_4 | \Phi \rangle &= 1 \end{aligned} \tag{15}$$

which means that the expected number of photons in each arm does not depend on the phase ϕ that is introduced in one path of the MBI, while the coincidence counts oscillate with ϕ.

In the second step, we consider the case of a coherent input state. In our experiment, the attenuated laser is linearly polarized and thus the initial quantum state for the MBI is $|\alpha\rangle_1 |0\rangle_2$. Remember, that a coherent state $|\alpha\rangle$ can be expressed as

$$|\alpha\rangle = e^{\frac{|\alpha|^2}{2}} \cdot \sum_{n=0}^{\infty} \frac{\alpha^n}{\sqrt{n!}} |n\rangle = e^{\frac{|\alpha|^2}{2}} \cdot e^{\alpha \hat{a}^\dagger} |0\rangle \tag{16}$$

Eq. (11) implies the relation

$$\hat{a}_1^\dagger = \frac{1}{2} \left(\left(e^{-i\phi} + 1 \right) \cdot \hat{a}_3^\dagger + \left(i e^{-i\phi} - i \right) \cdot \hat{a}_4^\dagger \right) \tag{17}$$

Therefore, we can rewrite our coherent quantum state as

$$\begin{aligned} |\Phi_{\mathrm{coh}}\rangle &= e^{\frac{|\alpha|^2}{2}} \cdot e^{\alpha \hat{a}_1^\dagger} |0\rangle = e^{\frac{|\alpha|^2}{2}} \cdot e^{\frac{\alpha}{2}(e^{-i\phi}+1)\cdot \hat{a}_3^\dagger} \cdot e^{\frac{\alpha}{2}(ie^{-i\phi}-i)\cdot \hat{a}_4^\dagger} |0\rangle \\ &= \left| \frac{\alpha}{2} \cdot \left(e^{-i\phi} + 1 \right) \right\rangle_3 \left| \frac{\alpha}{2} \cdot \left(i e^{-i\phi} - i \right) \right\rangle_4 \end{aligned} \tag{18}$$

We can now calculate the mean photon number in each arm, recalling that $\hat{n}\, |\alpha\rangle = |\alpha|^2 |\alpha\rangle$

$$\begin{aligned} \langle \Phi_{\mathrm{coh}} | \hat{n}_3 | \Phi_{\mathrm{coh}} \rangle &= |\alpha|^2 \cos^2\left(\frac{\phi}{2}\right) \\ \langle \Phi_{\mathrm{coh}} | \hat{n}_4 | \Phi_{\mathrm{coh}} \rangle &= |\alpha|^2 \sin^2\left(\frac{\phi}{2}\right) \end{aligned} \tag{19}$$

And the mean number of coincidences

$$\langle \Phi_{\mathrm{coh}} | \hat{n}_3 \hat{n}_4 | \Phi_{\mathrm{coh}} \rangle = \frac{|\alpha|^2}{4} \sin^2(\phi) \tag{20}$$

As shown in the experiment, the coincidences oscillate with double the frequency of the single arm counts.

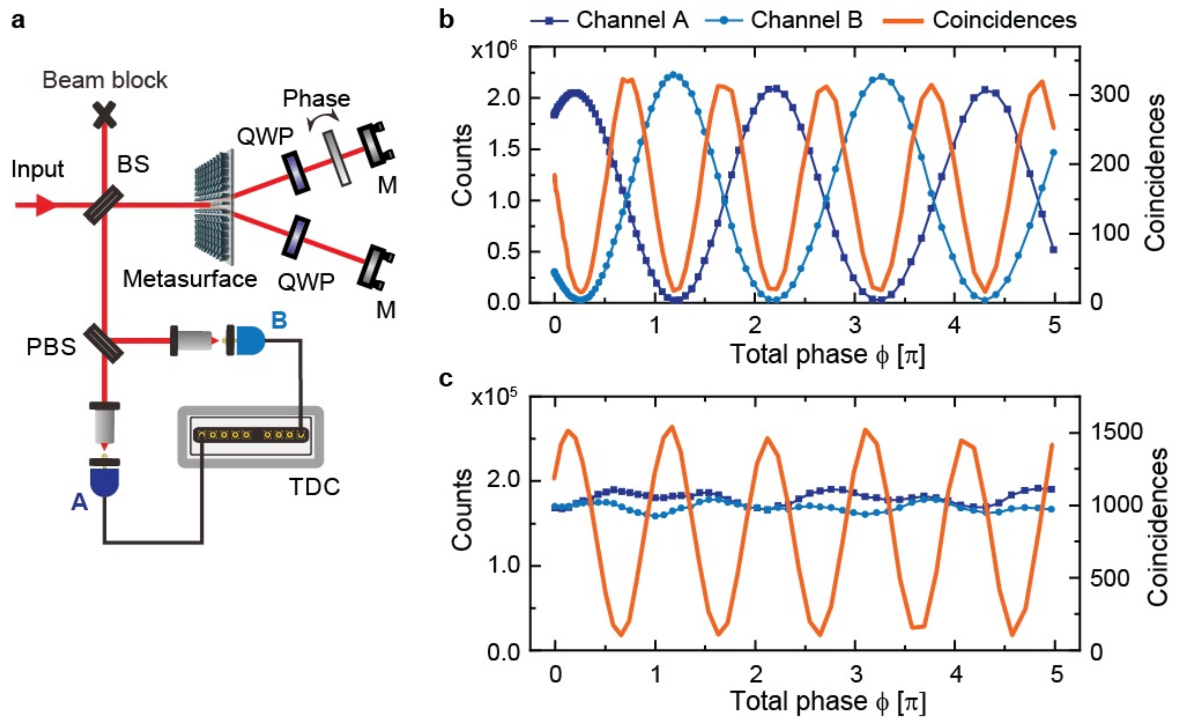

Fig. S2. Photon pair entanglement and disentanglement in a metasurface-based interferometer. a, Experimental setup based on a folded metasurface-based interferometer (MBI) for testing the coherence preservation at the metasurface. After the light passes the metasurface it is reflected back in both channels for a second interference. A thin glass plate introduces a variable phase difference ϕ between the two optical paths. A beam splitter (BS) and a polarizing beam splitter (PBS) are used to spatially separate the two MBI output channels, which are characterized by their linear polarization state. b, Experimental results for a weak coherent input state (classical state). The count rates at the detectors in both channels A and B show an oscillating behavior with the introduced phase ϕ with the highest coincidence rate when both detectors have balanced rates. c, Experimental results for the two-photon state of the SPDC source. The count rates at both detectors are independent of the introduced phase ϕ and only the coincidence rate shows the oscillatory behavior.

3. Quantum interferometer for delayed photons

An obvious question regarding our metasurface interferometer is how the time overlap of both incoming linearly polarized photons influences the measurement results. In Figure 4, we investigate this issue for different time delays. The main difference in these measurements is a lower visibility in the coincidence curve for higher time delays.

One can understand this behavior by considering the case of a high temporal delay, where the two photons interfere independently. In that case, each photon passes the interferometer independently. When we rewrite the quantum state as a function of the output modes, we have to keep the input indices to consider the distinguishability of the two incoming photons:

$$\begin{aligned} |\Phi_{\tau\to\infty}\rangle &= \hat{a}_1^\dagger \hat{a}_2^\dagger |0\rangle \\ &= \frac{1}{2}\left(\left(e^{-i\phi}+1\right)\cdot\ \hat{a}_{3,1}^\dagger + \left(ie^{-i\phi}-i\right)\ \cdot \hat{a}_{4,1}^\dagger\right) \\ &\cdot\left(\left(-ie^{-i\phi}+i\right)\cdot\ \hat{a}_{3,2}^\dagger + \left(e^{-i\phi}+i\right)\cdot\ \hat{a}_{4,2}^\dagger\right)|0\rangle \end{aligned} \tag{21}$$

For the expected photon number at each detector, we have to sum up the average photon number at the detector for both initial photons.

$$\begin{aligned} \langle\Phi_{\tau\to\infty}|\hat{n}_3|\Phi_{\tau\to\infty}\rangle &= 1 \\ \langle\Phi_{\tau\to\infty}|\hat{n}_4|\Phi_{\tau\to\infty}\rangle &= 1 \end{aligned} \tag{22}$$

For the coincidences, we have to sum up all cases that achieve coincidence counts. This means that either photon 1 has to go to channel 3 and photon 2 has to go to channel 4 or vice versa:

$$\langle\Phi_{\tau\to\infty}|\hat{n}_3\hat{n}_4|\Phi_{\tau\to\infty}\rangle = \frac{1}{4}\cdot\left(\cos(2\phi)+3\right) \tag{23}$$

Compared to the case of no time delay, these calculations still lead to constant photon rates at each single detector and to an oscillating behavior in the coincidences. However, the theoretical value for the visibility in this oscillation significantly drops from 100% to 33.3%. This trend coincides with our measurement results in Fig. 4 (from 86.6% to 44.5%).

4. Metasurface design and properties

The dielectric metasurface consists of etched silicon nanofins placed on a glass substrate. For the design, we choose the Pancharatnam-Berry-phase (PBP) concept, in which the local phase only depends on the assigned nanofin orientation. This geometric design concept is favorable since it ensures clean circular polarization states in the first diffraction orders regardless of the chosen design parameters and the related sample efficiency.

To achieve sensible fabrication parameters for the metasurface, we simulate a single fin with the rigorous coupled-wave analysis (RCWA) method with periodic boundary conditions. Contrary to the produced gradient phase field, this simulation does not account for rotational interactions between neighboring fins. However, for sufficiently large distances between the nanofins and only small phase changes along the surface, this effect might be negligible.

The fundamental principle of a PBP metasurface is its local half wave plate (HWP) functionality. Therefore, we chose a quadratic unit cell since it has a stronger rotational invariance compared to a rectangular shape. This leaves us with four degrees of freedom in the design of the nanofins (Figure S2): the periodicity p, the nanofin height h, the nanofin length l, and the nanofin width w.

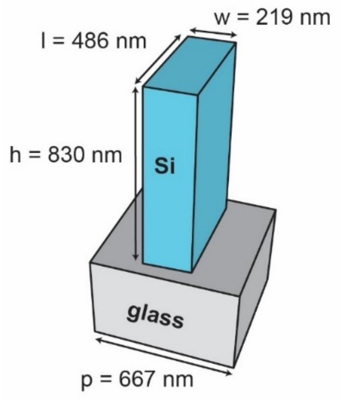

Figure S2. Simulated geometry for the optimization process. The structure has four degrees of freedom. We neglected the reflection on the backside of the glass substrate. For the simulation, we assumed a refractive index of $n_{Si} = 3.344 + i \cdot 5.1 \cdot 10^{-6}$ for the silicon nanofins and of $n_{glass} = 1.5$ for the substrate at a wavelength of 1550 nm.

For an efficient metasurface, these parameters have to be chosen in such a way, that the cross-polarization transmission efficiency T_{cross} is maximized:

$$T_{cross} = |\langle L|\hat{t}|R\rangle|^2 = \frac{1}{4} \cdot \left|t_{xx} - t_{yy} + i \cdot \left(t_{xy} + t_{yx}\right)\right|^2 \quad (24)$$

with the transmission matrix

$$t = \begin{pmatrix} t_{xx} & t_{yx} \\ t_{xy} & t_{yy} \end{pmatrix} \quad (25)$$

The values of the transmission matrix are obtained for normal incidence in the zeroth-order by the RCWA simulation. The parameter space with four degrees of freedom is very large for a parametric sweep approach. Therefore, we explored the entire search space first with a random search on 30,000 points and afterwards perform a local optimization with the Nelder-Mead algorithm. Thereby, we used the best results from the random search to generate the initial simplex for the Nelder-Mead algorithm.

To ensure that the optimized design is practical for fabrication, we forced a minimal gap distance between fins during the optimization process. Additionally, we averaged the results over four different fin orientations, to ensure that the half-wave plate functionality is not strongly orientation dependent.

After the optimization process, the calculated cross transmission reached 99.6% for the optimized design (see Figure S3). Note that this value neglects the reflectance on the backside of the substrate, which can be estimated with Fresnel as a 4% loss. Recent research already showed that dielectric metamaterials made of silicon can achieve very high efficiencies [1, 2]. Our simulations indicate that these high efficiencies are also achievable in our case with the Pancharatnam-Berry phase concept.

Since the efficiency peak in the calculated spectrum is reasonably broad, we expect that the design works within the fabrication tolerances.

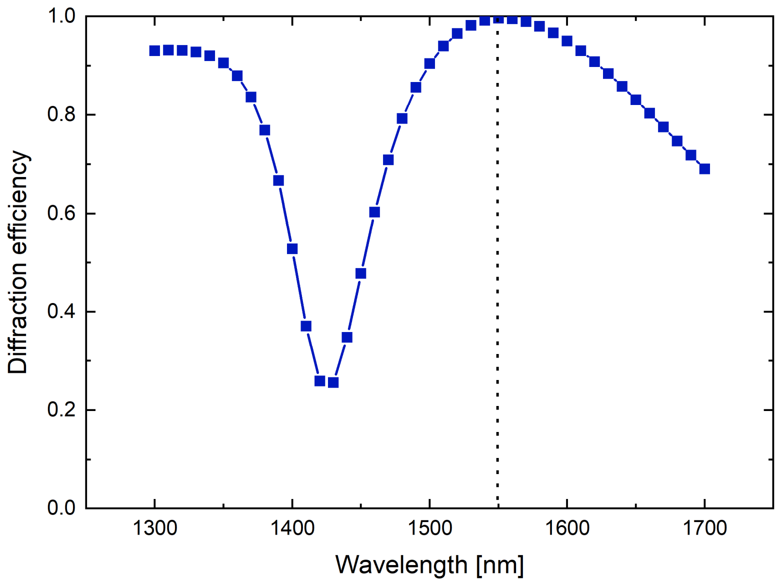

Figure S3. Simulated cross-polarization transmission efficiency for the optimized design at 1550 nm wavelength (dotted line). The evaluated cross transmission efficiency reach up to 99.6 % compared to the incident light. Note that the reflection on the backside of the glass substrate is not considered in this simulation.

For the fabrication of our metasurface, we used the size parameters of our optimized design. The dielectric silicon metasurface was fabricated on a glass substrate following the processes of deposition, patterning, lift-off, and etching. First, an 830-nm-thick amorphous silicon film was deposited by plasma enhanced chemical vapor deposition (PECVD). A poly-methyl-methacrylate (PMMA) resist layer was spin-coated on the silicon film and baked on a hotplate at 180 °C for 2 min to remove the solvent. Then the desired structure was patterned by standard electron beam lithography and subsequent development in 1:3 methylisobutylketon(MIBK):isopropanol(IPA) solution. Next, the sample was coated with a 45-nm-thick chromium layer by electron beam evaporation and a lift-off process in hot acetone was performed. Finally, the desired structure was transferred from chromium to silicon by using inductively coupled plasma reactive ion etching (ICP-RIE). The final metasurface field has a circular shape with a diameter of 300 µm. The silicon nanofins possess an 830nm height, a 486nm length, a 201 nm width, and are arranged in a rectangular grid with 667 nm period.

Figure S5 shows a scanning electron microscopy (SEM) image (top view) of the fabricated sample. The fabricated fins are homogenous in size without large variation that matches the parameters from the design (Table S1). From the SEM side view, we find that our etching process is quite anisotropic since the fin walls seem to be nearly vertical (see Fig. 2b in the main manuscript).

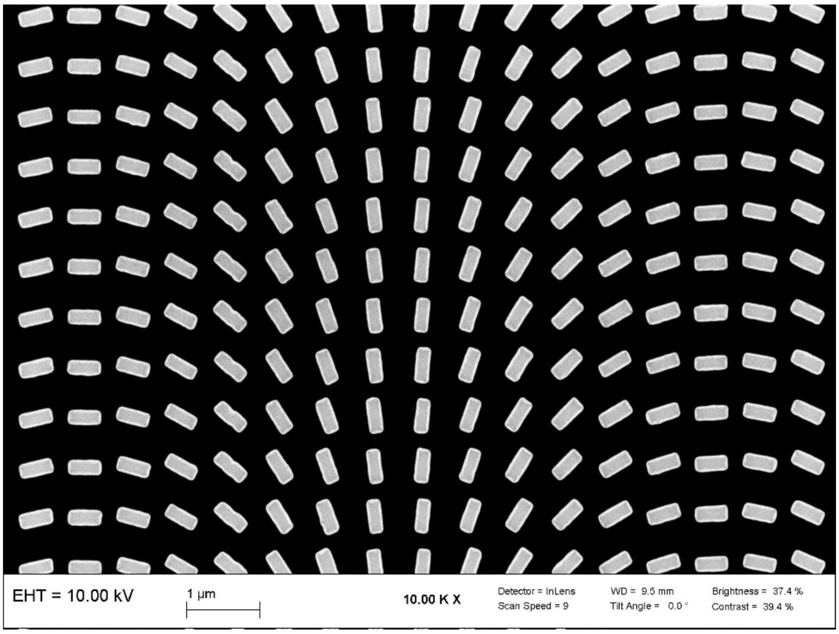

Figure S5. Scanning electron microscopy image of the fabricated metasurface sample generating a linear phase gradient in the horizontal direction.

Structural dimension	Optimized design	Fabricated
Width (nm)	219	201±5
Length (nm)	486	486±5

Table S1. Geometrical target and fabrication sizes of the silicon nanofins. The fabrication values are extracted from the SEM image (Figure S4).

For benchmark purposes, we measured the diffraction efficiencies of the sample over a large range of wavelengths (Figure S6). The measurement was done with the light from a tuneable OPA laser system with short laser pulses. Note that the measured efficiency spectrum is broadened due to the spectral bandwidth of around 50 nm for the laser pulses. We obtain the maximal absolute diffraction efficiency near the design wavelength of 1550 nm with a value of 42%.

Compared to our simulations, this value is considerably smaller. Notable in this regard is the huge discrepancy between the relative and the absolute efficiency values. This discrepancy appears since a considerable amount of the light is lost in reflection.

It is difficult to pinpoint the exact cause of these losses, but there are many possible explanations. For the simulation, we used a periodic arrangement of identical nanofins (all with the same orientation) to optimize the polarization conversion efficiency for an incoming plane wave. On the contrary, the experimental gradient structure breaks this assumed periodicity and the used Gaussian beam does possess non-perpendicular components. Both differences can affect the diffraction efficiency.

Even more critical are the assumptions regarding the refractive indices. For example, for silicon, we used the values measured in an ellipsometry setup on a thin film. However, the material is changing its internal state from amorphous to polycrystalline during the fabrication process depending on process time and temperature. The crystallinity influences the refractive index. Hence, the optimal structure design might be slightly different.

Moreover, the most critical source of error is usually the fabrication process itself. Variation in sizes, rough surfaces, anisotropic etching, and unwanted oxidation can all reduce the overall efficiency of the sample.

Although the experimental efficiency is lower than in our design, our results show that quantum interference is still possible.

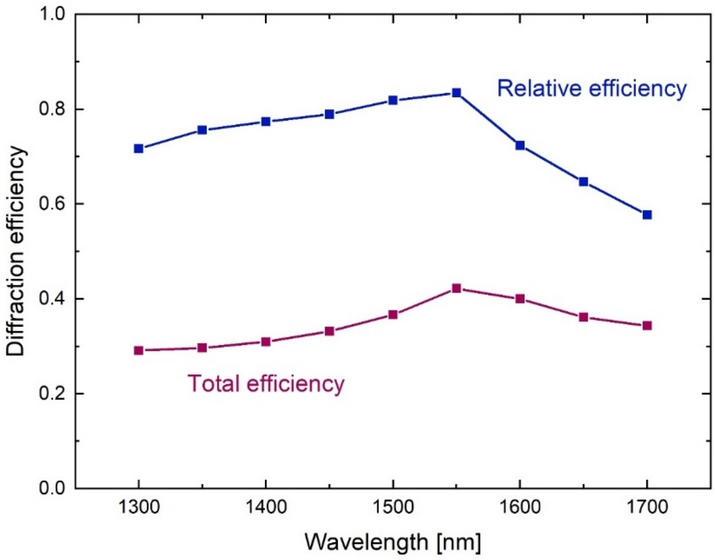

Figure S6. Experimentally determined diffraction efficiency for the transmitted light into the first diffraction orders (sum of -1st and 1st). At the design wavelength of 1550nm, our sample diffracts 83% of the transmitted light into the first diffraction orders (relative efficiency) and 42% of the total input light (absolute efficiency).

5. Stokes parameter characterization

The used Pancharatnam-Berry phase is based on the conversion of the circular polarization states of light. However, since the underlying symmetry arguments lose their validity for high diffraction angles, we verified the polarization state in the output state after the metasurface with a Stokes parameter measurement method, which is described by Schaefer et. al.[3] For this measurement method, we placed a quarter wave plate (QWP), a linear polarizer (LP) and a power meter in diffraction arm of the metasurface and measured under different QWP-angles (see Figure S6). From these data, we calculate the normalized Stokes vector for our metasurface:

$$S = \begin{pmatrix} 1 \\ -0.08 \\ -0.06 \\ 0.98 \end{pmatrix} \tag{26}$$

Note, that the last element of the Stokes vector S_3 is very close to one, which would correspond to perfectly right circularly polarized light ($S_{RCP} = (1,0,0,1)^T$).

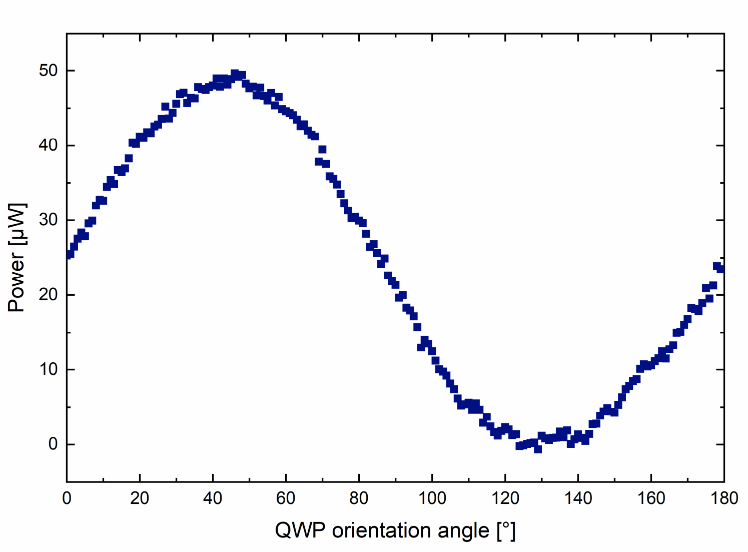

Figure S6. Stokes measurement for one metasurface channel. For the QWP-orientation of 45° and 135°, we project on the two circular polarizations respectively which leads to the corresponding minimum and maximum of the measured oscillation curve.

6. Reference setup and photon pair generation

The Type-II SPDC source that is used in our experiment is based on a 35 mm long titanium indiffused lithium niobate ($LiNbO_3$) waveguide (Figure S7). The source is engineered to produce pairs of photons centered around a wavelength of 1550 nm. To ensure indistinguishability, the source is pumped with a cw-laser with 0.5 mW at 775.4 nm wavelength. Furthermore, its temperature is kept constant at 30°C. This produces pairs of photons with the orthogonal linear polarization that are subsequently routed to the Michelson interferometer via polarization maintaining fibers (PMFs).

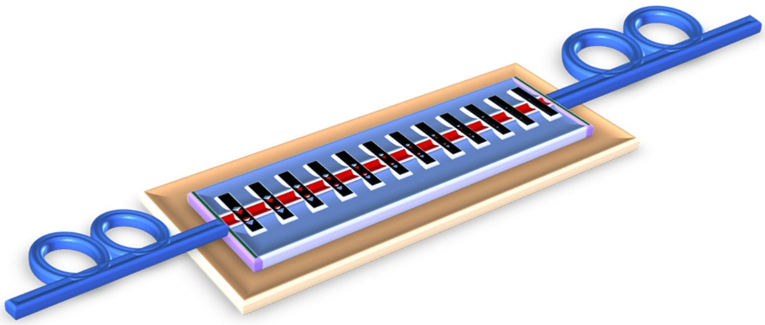

Figure S7. Schematical illustration of the two-photon source (various components not to scale). The $LiNbO_3$ crystal (blue shape) lies on a copper holder (brown) which is temperature stabilized. The red section represents the Titanium indiffused waveguide, across which one can see a schematic representation of the poling pattern (black stripes). The blue loops represent polarization-maintaining fibers which are directly pigtailed with UV glue to the end-faces of the waveguide.[4] The chip brightness of two-photon generation was measured to be around (1.39 ± 0.04) $\times 10^7$ pairs/(s · mW· nm).

Optical dispersion in the PMFs and the $LiNbO_3$ crystal itself will decrease the temporal overlap of the photons in the two polarization states. However, the Michelson interferometer can compensate for the delay time between the two photons, since the two photons are separated with the polarizing beam splitter and a different temporal delay is applied to only one polarization state. The transverse beam shape is Gaussian and focused to a spot diameter of 190 µm at the metasurface. In the experiment, we use regular lenses in front and behind the metasurface to adapt the beam size to the metasurface field size of 300 µm. Thus, after passing the metasurface and an additional lens, the beam is re-collimated and beam diffraction is a negligible. In our experiments, we oriented the metasurface towards the incident beam.

Finally, the light is collected by coupling the two diffraction modes from our metasurface into single mode fibers (SMFs). A pair of WDM filters with 1.4 nm bandwidth centered at 1550 nm (one filter per arm) ensures proper noise suppression of secondary processes (e.g. fluorescence) that might pollute the results. The two filter outputs are routed into the SNSPDs (~90% efficiency at 1550 nm) for detection.

To put the performance of the metasurface in perspective, we carry out a calibration experiment for the SPDC source on its own. This experiment is inspired by Grice and Walmsley [5] and allows us to determine an upper bound on the achievable HOM visibility. Here, we use a half-wave plate to rotate the linear polarization states to the diagonal (D) and anti-diagonal (A) bases creating the quantum state $|D\rangle|A\rangle = \frac{1}{2}\left(\hat{a}_H^\dagger\hat{a}_H^\dagger - \hat{a}_V^\dagger\hat{a}_V^\dagger\right)|0\rangle$. This state is then inserted into a polarization fiber beam splitter, where the two photons interfere creating once again a two-photon NOON state (Figure S8). The reference experiment shows a HOM dip ($C_{45} + C_{46}$) with a visibility of $89 \pm 5\,\%$ and its corresponding "anti"-HOM peak (C_{56}) for the coincidences (Figure 3 in the main text). Note that a significant experimental difference between the metasurface and reference setup is the number of fiber coupling points. As a consequence, the coupling losses due to mirror tilting during transversal motor movement affects both channels differently for the metasurface setup. We assume that this is the main reason for the asymmetric features in the HOM-coincidence counts in Figure 3.

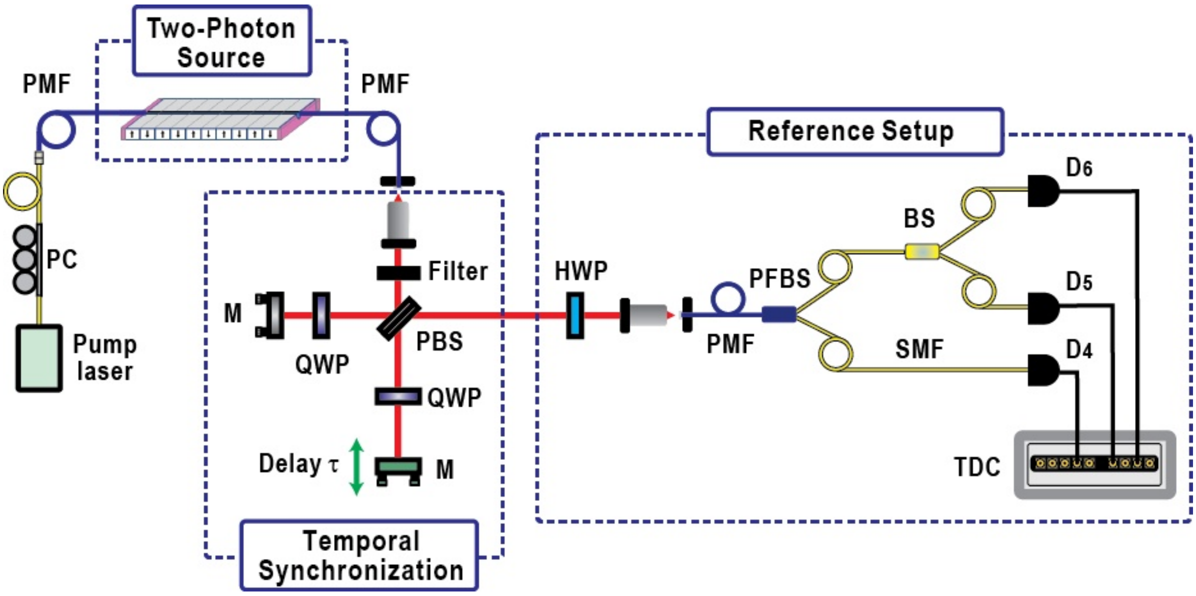

Figure S8. Schematical illustration of the reference setup for characterization of the SPDC source. The metasurface is replaced by a half-wave plate (HWP) and a polarizing fiber beam splitter (PFBS). In contrast to the metasurface, our reference setup performs a polarization basis change to the diagonal

basis, which leads to the generation of a NOON state, too. Therefore, it can be used as a benchmark for the quality of the initial quantum state. (PMF – polarization maintaining fiber, SMF – single mode fiber, M – mirror, PC – polarization controller, QWP – quarter-wave plate, PBS – polarizing beam splitter, BS – beam splitter, TDC – time-digital converter).

7. HOM data analysis

Each point in the scan of the time delay τ is the average of ten measurements and its associated error is the standard deviation of such average. An obvious ringing is visible in both coincidence plots (Figure 3) with a more pronounced effect near the dip. This is an effect due to the filters used in the detection arms, specifically the convolution of the filters top-hat function with the phase-matching sinc-function. This influences the data analysis when extracting the maximum visibility. In addition, a small drift due to a slight misalignment of the motor motion's axis is visible in the raw data. In order to compensate for this, a linear fit has been applied considering only the data away from the dip region. This "baseline" has been then used to normalize the whole dataset, ensuring a fair estimation of the visibility.

The visibility is defined as

$$V = \frac{I_+ - I_-}{I_+} \tag{27}$$

where I_+ and I_- are the maximum and minimum intensities registered in the coincidences, respectively. In our case, after normalization, these correspond to 1 and the minimum value of the normalized coincidences respectively, i.e.

$$V = 1 - \frac{C(\tau = 0)}{N(\tau = 0)} \tag{28}$$

where $C(\tau)$ and $N(\tau)$ are the values of the registered coincidences and the normalization baseline at time delay τ, respectively. Note, that the visibility of the interferometer (see Fig. 4) has a slightly different definition:

$$V_{interferometer} = \frac{I_+ - I_-}{I_+ + I_-} \tag{29}$$

8. References

1. Decker M, Staude I, Falkner M, Dominguez J, Neshev DN, Brener I, *et al.* High-efficiency dielectric Huygens' surfaces. *Advanced Optical Materials* 2015, 3(6): 813--820.
2. Arbabi E, Arbabi A, Kamali SM, Horie Y, Faraon A. High efficiency double-wavelength dielectric metasurface lenses with dichroic birefringent meta-atoms. *Optics Express* 2016, 24(16): 18468--18477.
3. Schaefer B, Collett E, Smyth R, Barrett D, Fraher B. Measuring the Stokes polarization parameters. *Am J Phys* 2007, 75(2): 163-168.
4. Montaut N, Sansoni L, Meyer-Scott E, Ricken R, Quiring V, Herrmann H, *et al.* High-efficiency plug-and-play source of heralded single photons. *Physical Review Applied* 2017, 8(2): 024021.
5. Grice WP, Walmsley IA. Spectral information and distinguishability in type-II down-conversion with a broadband pump. *Physical Review A* 1997, 56(2): 1627.
